# Array tomography: 15 years of synaptic analysis

**DOI:** 10.1042/NS20220013

**Published:** 2022-09-23

**Authors:** Anna Sanchez Avila, Christopher M. Henstridge

**Affiliations:** 1 Euan Macdonald Centre for Motor Neuron Disease, Edinburgh, UK; 2 Division of Cellular and Systems Medicine, University of Dundee, Dundee, UK

**Keywords:** ALS, array tomography, brain, synapses

## Abstract

Synapses are minuscule, intricate structures crucial for the correct communication between neurons. In the 125 years since the term synapse was first coined, we have advanced a long way when it comes to our understanding of how they work and what they do. Most of the fundamental discoveries have been invariably linked to advances in technology. However, due to their size, delicate structural integrity and their sheer number, our knowledge of synaptic biology has remained somewhat elusive and their role in neurodegenerative diseases still remains largely unknown. Here, we briefly discuss some of the imaging technologies used to study synapses and focus on the utility of the high-resolution imaging technique array tomography (AT). We introduce the AT technique and highlight some of the ways it is utilised with a particular focus on its power for analysing synaptic composition and pathology in human post-mortem tissue. We also discuss some of the benefits and drawbacks of techniques for imaging synapses and highlight some recent advances in the study of form and function by combining physiology and high-resolution synaptic imaging.

## Introduction

Throughout history, scientific advances are almost always tied to technological breakthroughs, and this holds true within the field of neuroscience. Ramón Santiago y Cajal used contemporary staining techniques in the late 1800s to highlight with exquisite neuroanatomical detail and draw the most beautiful – and anatomically accurate – images of brain cells and other biological structures [1,2]. Thanks to pioneering work in the early 1900s by neuroscientists like Otto Loewi [3,4], Henry Dale and Paul Gaddum [5], we now understand the chemical nature of synapses and how neurotransmitters control neuronal function. More recently, in the 1970s, Neher and Sakmann developed patch clamp electrophysiology [6,7], at the time a revolutionary technique that allowed the recording of single ion channel activity in neurons, helping researchers explore the fundamental processes that govern neuronal activity. Our more recent advances in understanding synaptic function are built upon many of these foundational studies and techniques.

All the machinery required for the generation, release, receipt and response to neurotransmitters is maintained within minute synaptic compartments. Incredibly, it is estimated we have more of these synaptic contacts in our brain than there are stars in our galaxy [8]. Given their astronomical number, it follows that their size would be in the nanoscale. This is why visualising synapses is incredibly difficult, yet essential, since form and function go hand in hand and understanding their structure can provide crucial information on how they work. However, it wasn’t until the invention of the electron microscope in the mid 1900s that we could fully appreciate the intricacy of a synapse.

In 1955, the first electron microscopy image of a vertebrate’s central nervous system synapse was published [9]. Electron microscopy uses a controlled beam of electrons rather than light to visualise the sample, which means resolutions in the low tens of nanometres can be achieved. This technique helped increase our knowledge of synapse structure and allowed researchers to visualise critical synaptic components such as presynaptic vesicles and the post synaptic density for the first time. Electron microscopy remains the gold standard technique for studying synaptic anatomy. The two main electron microscopy modalities are transmission electron microscopy (TEM) and scanning electron microscopy (SEM). Essentially TEM relies on electrons travelling through ultrathin sample sections to build a monochrome 2D image, whereas SEM detects the electrons as they rebound off the sample providing a 3D image of the sample surface. The use of TEM or SEM will depend on the kind of information you want to gather from your sample and for a more detailed description of their sample requirements and differences in image capture, there are several comprehensive reviews on the topic [10,11]. Retaining the synaptic ultrastructural integrity throughout the sample processing can be challenging. It is also worth noting that electron microscopy is labour intensive which usually restricts the study to only a relatively small number of synapses at one given time, as well as being quite technically demanding and requiring expensive, specialised equipment. Nonetheless, electron microscopes combined with block-face trimming can automate the process of imaging large blocks of cortex at a synaptic level. This provides invaluable data on neuronal connectivity and synaptic architecture, but the amount of data generated are difficult to handle which means this approach is often restricted to large institutes and consortia employing many research staff, such as The Machine Intelligence from Cortical Networks (MICrONS) program at the Allen Brain institute, who are using TEM and artificial intelligence to reconstruct 200,000 cortical cells and 500,000,000 synapses [12,13]. Another handicap is the fact that labelling proteins of interest using immuno-EM techniques is often hindered by poor antibody penetration due to resin embedding. This can sometimes be bypassed by performing post-embedding immuno-EM, but not all antibodies are suitable for this approach and rigorous controls are needed. Non-specific labelling, resin embedding issues and an overall lack of suitable antibodies, means studies of synaptic protein composition can prove difficult using electron microscopy [14]; therefore, we need complimentary techniques that overcome the inescapable drawbacks.

**Figure 1:**
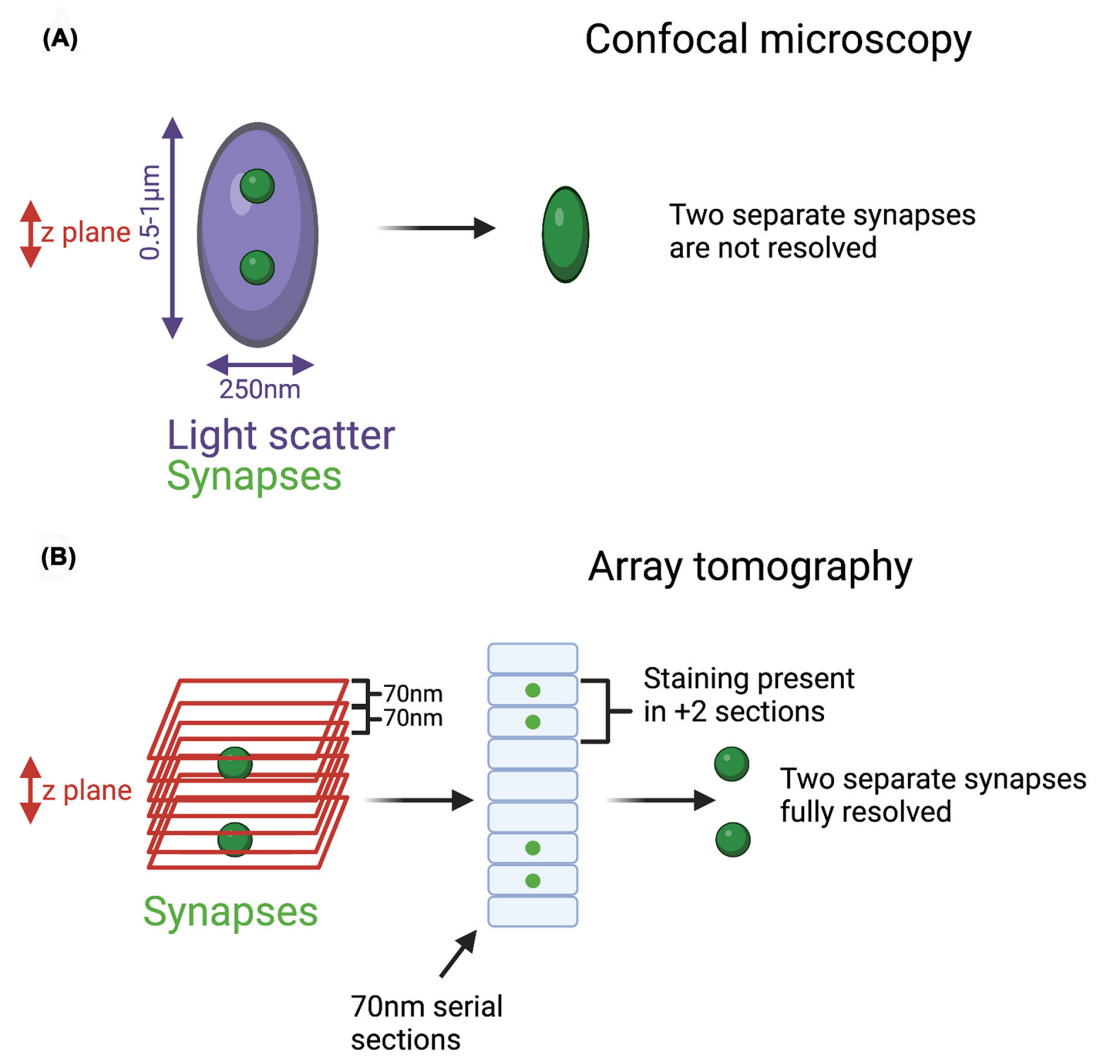
Array tomography: improving axial resolution by physical dissection (**A**) Due to the limitations of confocal resolution and light scatter within tissue, some synapses within a 3D space will not be resolved, as their fluorophore emission will overlap and be recorded as one large puncta. (**B**) AT addresses this problem by physically sectioning the tissue at 70 nm. This significantly improves axial resolution from 500–1000 to 70 nm. Synapses are larger than 70 nm, so real synaptic puncta should be present in several consecutive sections. Only collecting and analysing these 3D puncta allows for accurate single synapse identification. A summary of the processing can be found in Figure 2. Created with BioRender.com.

Fluorescence-based light microscopy has advanced dramatically in recent decades, with the development of high-resolution and super-resolution techniques. However with even the most powerful confocal microscope, it is difficult to visualise individual objects that are within approximately 250 nm of each other in the *x–y* plane. This is due to the unavoidable light diffraction limit which is determined by the microscope optics and the wavelength of light emitted by the detection fluorophore. Furthermore, mostly due to light scatter, the best confocal axial resolution (*z*-plane) within tissue is approximately 0.5–1 micron. Synapses are tightly packed into a dense neuropil and although deconvolved confocal imaging may distinguish synapses in two dimensions, it will not achieve the resolution required to observe single synapses accurately and consistently in 3D (Figure 1A). Therefore, imaging techniques that can break this diffraction limit are required to accurately identify individual synapses within intact tissue. Super resolution microscopy techniques were developed by the Nobel Prize winning collective of Eric Betzig, Stefan Hell and William E. Moerner, and have facilitated huge advances in our understanding of neuronal and synaptic composition [15]. However, although techniques such as STORM (STochastic Optical Resolution Microscopy), STED (STimulated Emission Depletion microscopy) and PALM (PhotoActivated Localisation Microscopy) can achieve resolutions beyond the diffraction limit, they are expensive, and require extensive optimisation and experienced users.

Essentially, the technique of choice comes down to the question being addressed. For instance, if the aim is to study synaptic protein composition at scale, mass spectrometry–based proteomics on synaptically enriched samples from fresh frozen tissue can identify thousands of proteins [16–18]. This technique has provided crucial information on the molecular structure of the synapse and revealed numerous disease-associated protein changes [18–20]. This approach also allows for protein quantification, but at the cost of any intact tissue context as the entire sample is homogenised before analysis. Obviously, this also prevents any analysis of synaptic density or anatomy. Ideally, in many circumstances, it would be useful to analyse multiple proteins in individual synapses, in intact tissue, and in a high-throughput manner. Furthermore, from a disease context, the reliance on animal models has proved somewhat unreliable for impactful translational output, and arguably, a greater characterisation of human post-mortem material will provide greater insight into the molecular mechanisms driving neurodegenerative disease. Given the critical role of synapses in normal brain function and their early breakdown in neurodegenerative disease, it is important we have the tools to study molecular changes within identifiable synaptic populations, to unravel the molecular mechanisms driving synapse loss.

Here, we will introduce the high-resolution imaging technique array tomography (AT) which allows multiplexed analysis at the single synapse level of human brain and summarise its impact on synaptic biology over the past 15 years.

## Array tomography

**Figure 2:**
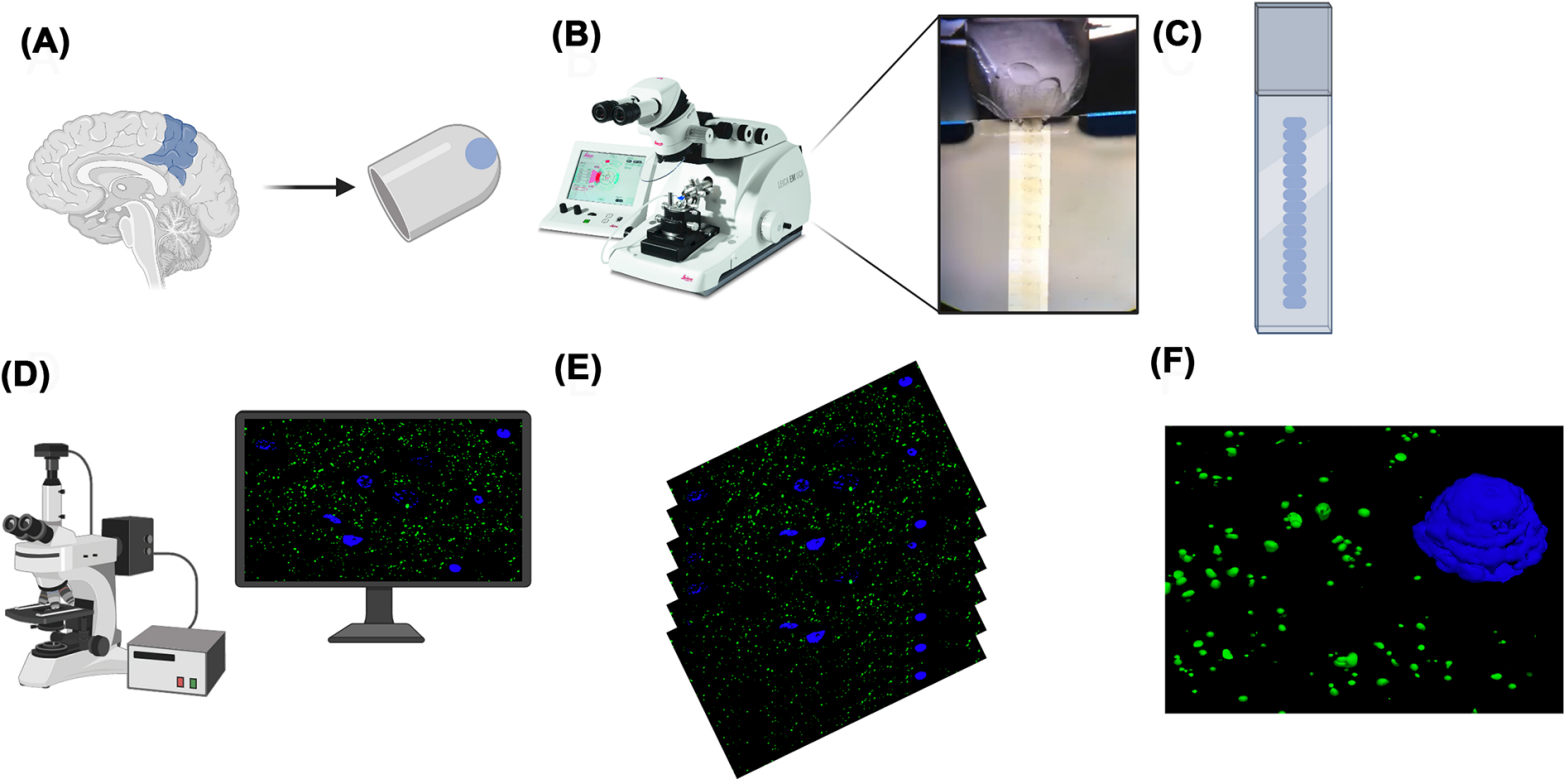
Array tomography workflow summary (**A**) Tissue is obtained from fresh post-mortem brain, lightly fixed in 4% PFA and then embedded in an LR White resin capsule. (**B**) Blocks are cut into approximately 20 serial sections of 70 nm thickness using an ultramicrotome, and the ribbon is collected on to gelatin-coated coverslips. (**C**) Ribbons are blocked and stained using standard immunostaining protocols and mounted on to a slide. (**D**) Images are taken using a widefield epifluorescence microscope equipped with a 63× 1.4NA Plan Apochromat objective. (**E**) Twenty consecutive images are taken at the same position along the ribbon and then stacked into 3D volumes ready for analysis. (**F**) Post-hoc 3D rendering of pre-synapses (synaptophysin, green) and nuclei (DAPI, blue). Created with BioRender.com

Since its development in 2007 [22] and its later adaptation for use in human post-mortem tissue [14], the high-resolution imaging technique known as immunofluorescent AT has been an invaluable tool for studying synapses at both a density analysis and protein composition level, without the need for highly specialised microscopes. AT overcomes the axial resolution problem of light microscopy by the simple – yet effective – approach of physically dissecting the tissue in ultrathin (70 nm) serial sections of resin-embedded post-mortem tissue (Figures 1B and 2A–C). This technique is not to be confused with SEM AT, which applies a similar ultrathin dissection approach but uses an EM to visualise the sample and post-hoc 3D rendering. The serial AT sections – also called ribbons – can then be stained using standard immunofluorescence protocols and the antibodies can be stripped and the sample can be re-stained [23,24]. This already proves an advantage over electron microscopy as it allows multiplexing of several proteins [25], and it avoids the use of heavy metals, making it a safer, more accessible technique. Multiplexing is a huge advantage of AT, allowing the analysis of multiple proteins within the same individually resolved synapses. Theoretically, this process can be performed repeatedly; however, multiple rounds of washes and buffers eventually take their toll on the tissue. Extensive validation is required to ensure antibodies are efficiently stripped; otherwise, contamination of previous staining can influence the interpretation of future staining rounds. Over repeated strips, tissue quality degrades and staining performance drops; however, this approach has yielded good quality imaging over four staining rounds of pathological amyloid proteins in the brain of a mouse model of Alzheimer’s disease [26] and allowed the identification of 18 synaptic markers for stratifying excitatory and inhibitory synapses over 6 rounds of staining and imaging [25]. The images can be obtained using a widefield epifluorescence microscope, which is more convenient than EM and super resolution techniques [23]. AT is accessible to many research teams with access to a few key pieces of equipment, often readily available in many imaging facilities.

**Figure 3:**
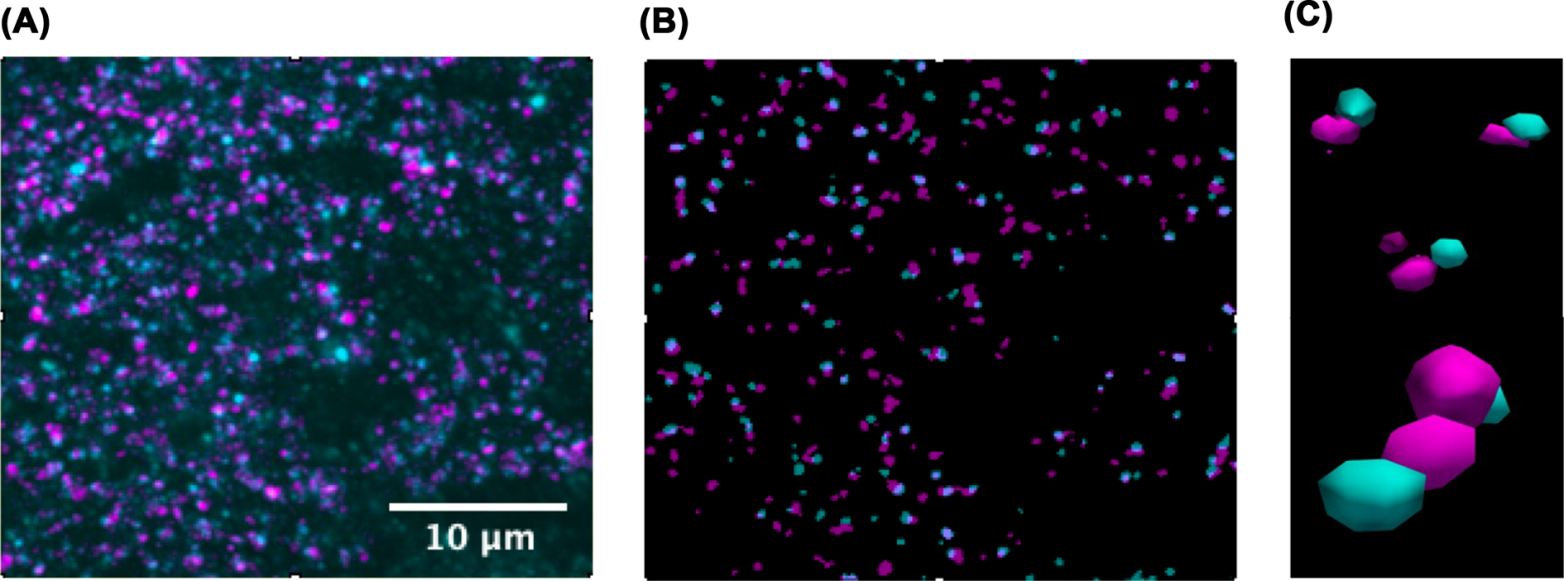
Basic image editing workflow (**A**) Example of a stacked AT image, consisting of 20 serial 70 nm sections. Synaptophysin (presynapse) in magenta and PSD95 (postsynapse) in cyan. (**B**) Example of the same image after segmentation, which removes background puncta present in only one 70 nm section and binarizes the retained synaptic puncta which are present in at least two consecutive sections. (**C**) Example of 3D-rendered synapses from (B). See the close and direct apposition of the re and post-synaptic markers.

The analysis performed after image acquisition will vary depending on the question being investigated, but all AT analyses begin with 2D alignment of the serial images. Once the images are accurately aligned, they are rendered into 3D image stacks. These steps can be performed successfully with image alignment tools in open-access platforms such as FIJI, but many labs are now writing their own scripts for automating batch alignments and downstream analyses. For example, we perform synapse density analysis and protein co-localisation analysis on segmented images (Figure 3A,B) with freely available MATLAB scripts [27,28] and use open access visualisation software for 3D rendering [29] (Figure 3C). Essentially, the script cleans up the images by removing puncta only found in one section (most likely background) and retaining 3D puncta. These are segmented and binarized, ready for counting. Several imaging channels can be prepared in the same way and co-localisation of proteins at the single synapse level, quantified. Ultimately, the goal is to accurately segment and quantify true synaptic puncta in 3D and assess the co-localisation of target proteins. More complex custom-made scripts have been generated to accurately identify synapse subtypes and even subsynaptic composition, using a combination of closely opposed pre and postsynaptic proteins [30]. The researchers called this approach SubSynMAP (SUB-SYNaptic, Multiplexed Analysis of Proteins). As research teams start tackling larger 3D volumes, the sheer size of the image files and analysis demands will require significant hardware performance and storage capabilities. Likely researchers will naturally navigate towards cloud-based software and machine learning approaches to efficiently process and analyse the vast amounts of imaging data.

We have briefly summarised our AT protocol below (Figure 2A–F), but more details can be found in Kay et al., 2013 [14] and in the excellent and comprehensive review by one of the inventors of the technique, Prof. Stephen Smith [23].

## Utility of array tomography

In most neurodegenerative diseases, such as Alzheimer’s, Parkinson’s, Huntington’s and motor neuron disease, synapse degeneration is thought to occur prior to neuronal loss [31]. This suggests synapses are vulnerable in disease, but importantly we know they are also extremely plastic, making them an exciting avenue for therapeutics to slow or even stop disease progression. However, there is still a lot to learn about the underlying mechanisms driving synaptic pathology, which is why having different imaging tools in our arsenal will be essential in making those advances.

AT has been used as a high-resolution imaging technique to study synapses in several disease contexts, such as Alzheimer’s disease, Fragile-X syndrome (FXS) and amyotrophic lateral sclerosis (ALS) [26,32–38]. Dr Kristina Micheva, one of the pioneers of this technique along with Prof. Stephen Smith, has recently used it to study the involvement of different synapse subtypes in FXS [38]. In FXS, increased tortuous spine density is well-characterised at an anatomical level in mouse models, but the molecular identity of these changes has been difficult to discern [39,40]. They revealed a layer-specific increase in small VGluT1 positive synapses in the somatosensory cortex and a decrease of large VGlut1-positive synapses, as well as a decrease of large inhibitory synapses throughout all cortical layers, in a model of FXS. Interestingly, they also found a change in the astrocytic association with excitatory synapses. This level of spatial and molecular detail at this scale would have proven extremely difficult with other imaging approaches and really showcases the power of single-synapse, multiplex imaging of intact tissue.

Prof. Tara Spires-Jones was one of the first neuroscientists to utilise the power of AT for the analysis of human post-mortem brain. Her work has led to significant advances in our understanding of several mechanisms involved in synaptic pathology in Alzheimer’s disease. For example her team have described the presence of β-amyloid [26,41], and Tau in Alzheimer’s synapses [26,35,42–45] as well as the synaptic presence of α-synuclein, tau and β-amyloid in synapses of dementia with Lewy bodies brains [33,46]. More recently, they have also used AT to reveal loss of inhibitory synapses near amyloid plaques in Alzheimer’s disease and an accumulation of amyloid in remaining synapses [41]. Furthermore, her team have also begun an important programme of work exploring synaptic changes within a healthy-ageing cohort [47,48].

It is important to note that other groups have used this technique to study several aspects of synapse biology in different contexts. Granger et al. studied the localisation of several synaptic proteins in the pre-synapse of ChAT^+^ and VIP^+^ interneurons in the mouse cortex [49]. AT has also been used to understand the synaptic connectome, for instance Bloss et al., saw that presynaptic axons form clusters of synapses on the distal dendrites of CA1 pyramidal neurons in mouse hippocampus [50]; moreover, Rah et al., used AT to assess synaptic input to the layer 5 pyramidal neurons in mouse somatosensory cortex [51].

As a research group, we are interested in the association between synapse degeneration and clinical presentation of ALS. Briefly, ALS is a devastatingly fatal neurodegenerative disease primarily affecting motor neurons, which leads to muscle weakness, difficulty swallowing and talking, and eventually inability to breathe (which is usually the cause of death, occurring 2–5 years after diagnosis). Up to 50% of ALS patients will also display some level of cognitive or behavioural impairment, which is very important as these patients have a worse prognosis, and we still do not understand the underlying pathological correlate [52–54].

In ALS, motor neurons die. Two of the main ideas as to why that happens are the die back versus die forward hypotheses. The die back hypothesis postulates that, following a toxic insult, there is synaptic degeneration, which leads to axonal retraction and motor neuron death [55–57]; whereas the die forward hypothesis centres on excitotoxicity, claiming a hyperexcitability of the motor neurons or an excess of glutamate leads to an increase of intracellular calcium, which is toxic to the cells therefore causing neuronal death [58–64]. It is important to highlight that both hypotheses place synapses at the core. In fact, synapse loss preceding motor neuron death has been found to occur both in the peripheral and central nervous system in ALS [65–69]. While working in the Spires-Jones lab, we used AT to show that synapse loss in the dorsolateral prefrontal cortex of ALS patients was also linked to cognitive impairments [34]. This was the first evidence that synapse loss is associated with the cognitive features of many ALS patients. AT allowed us to analyse approximately 45,000 synapses per sample, compared to 100 per sample by TEM. It also allowed us to show for the first time, that ALS-associated pathology was found at the human synapse.

Following this discovery, our interest lies in understanding the pathomechanisms underlying synapse degeneration in ALS, with a special focus in using human post-mortem tissue. We use AT to study potential links between synapse loss in different areas of the brain and symptoms displayed by patients, such as different types of cognitive or behavioural impairments. We also use array tomography to study the potential synaptic localisation of ALS-associated proteins, namely, aggregation-prone proteins known to be associated with ALS such as TDP-43 (aggregates are found in up to 97% of cases [70], and mutations in TDP-43 are disease-causative [71]) or FUS (protein aggregates in ALS and mutations in gene are disease-causative [72–75]). To do so, we use AT to study the colocalisation of target proteins with either pre or post synaptic markers. This level of molecular detail is difficult to obtain accurately with other imaging methods unless you have the ability and resources to perform super-resolution microscopy.

While AT has facilitated great insight into synaptic composition and pathology in disease, there are caveats that currently prevent its mainstream use. Resin-embedding can mask epitopes and make some proteins difficult to visualise. Learning to use an ultramicrotome is time-consuming and a demanding technique that some will not master. Analysing and interpreting thousands of synaptic puncta in 3D can be challenging. However, all these caveats are surmountable and the rewards for persevering are significant. As the field advances and technology improves, it is interesting to see how the benefits of array tomography are incorporated to unlock their full potential and we will discuss some of these below.

## Recent advances in array tomography

AT can be combined with other imaging techniques in what’s called conjugate AT [50,76,77]. For example, careful sample processing allows the combination of AT with EM. The ultrathin tissue ribbons are immunostained and imaged as per conventional immunofluorescent array tomography protocols then the ribbons are washed and prepped for EM imaging. This technique consequently combines the anatomical insight of EM with fluorescent multiplexing of several proteins, providing both anatomical and molecular data at a single synapse resolution. The combination of different protocols can affect ultrastructure and immunostaining so currently only a very small number of labs have optimised this approach. As processing techniques improve, the power of combining these anatomical and molecular imaging modalities will be fully realised. Another development is the use of super-resolution imaging on array tomography ribbons. Recent work studying the amyloid composition within and around amyloid plaques in Alzheimer’s disease, reconstructed an entire amyloid plaque using confocal-array tomography and STED-array tomography [78]. The improved resolution and detection of smaller amyloid fragments when AT and STED were combined highlight this combination of imaging modalities as a powerful way to achieve super-resolution in all three dimensions. Also, array tomoghraphy has recently been combined with structured illumination microscopy (SIM) to reveal distinct cortical synaptic input from different areas of the thalamus in mouse brain [79]. The research team named this approach structured illumination microscopy on the putative region of interest on ultrathin sections (SIM-PRIUS). Combining super-resolution imaging and AT appears an attractive method for gaining 3D data at the nanoscale, but at present is not widely accessible due to the limitations of super-resolution imaging noted previously.

Some groups have combined the study of form and function by performing AT on physiologically characterised synapses [80–82]. They achieved this by filling neurons with a dye via their recording electrode, and then embedding the tissue in resin before performing AT. These studies provide exquisite detail on the synaptic connectivity between paired cells. Holderith et al. have devised an improved protocol for the imaging of physiologically characterised synapses in epoxy resin-embedded tissue [83]. This paper is an impressive resource, describing several advances in the utility of AT and an improved methodology. For instance, some epitopes can be blocked by the hard resin used for AT, yet this paper describes alternative tissue embedding and processing approaches, increasing the success rate for primary antibody binding. The ultrathin ribbons were etched with Na-ethanolate to improve antigenicity and could be labelled in multiple rounds, allowing for the multiplexing of proteins. They also performed STED microscopy on characterised synapses, gaining super-resolution in all three dimensions. To highlight the power of their optimised imaging protocol they describe its use following paired recordings, two-photon [Ca^2+^] or glutamate-sensor (iGluSnFR) imaging in slice preparations. They discovered that axonal varicosities in which Ca^2+^ release was not evoked by an action potential contained vGluT1 but did not contain Munc-13-1 or opposing PSD-95. This suggests these axonal swellings contain presynaptic vesicles but do not contain a presynaptic active zone and do not form functional synapses. A striking example of correlated function and molecular composition at a single synapse scale.

These new approaches allow us to closely correlate changes in synapse function (based on the electrophysiological recordings) with changes in their structure or protein composition. As technology develops, the high-resolution scrutiny of physiologically characterised synapses is becoming more attainable, opening many avenues of research, and providing crucial understanding of synaptic biology. Furthermore, these studies highlight AT as a powerful yet accessible imaging tool that can be utilised to provide form to functional readouts. Reflecting on how these 125 years have shaped and shifted our understanding of the synapse, we can't help but wonder what Sir Sherrington would say if he were able to see the beautiful images of synapses in the fantastic detail that AT provides.

## Open Access

Open access for this article was enabled by the participation of University of Dundee in an all-inclusive Read & Publish agreement with Portland Press and the Biochemical Society under a transformative agreement with JISC.

## Abbreviations

ALS: amyotrophic lateral sclerosis
AT: array tomography
FXS: Fragile-X syndrome
SIM: structured illumination microscopy
SEM: scanning electron microscopy
TEM: transmission electron microscopy
